# Correction: Accurate predictions of the electronic excited states of BODIPY based dye sensitizers using spin-component-scaled double-hybrid functionals: a TD-DFT benchmark study

**DOI:** 10.1039/d5ra90089a

**Published:** 2025-07-28

**Authors:** Qabas Alkhatib, Wissam Helal, Ali Marashdeh

**Affiliations:** a Department of Chemistry, The University of Jordan Amman 11 942 Jordan wissam.helal@ju.edu.jo; b Department of Chemistry, Al-Balqa Applied University 19 117 Al-Salt Jordan; c Leiden Institute of Chemistry, Gorlaeus Laboratories, Leiden University P. O. Box 9502 2300 RA Leiden The Netherlands

## Abstract

Correction for ‘Accurate predictions of the electronic excited states of BODIPY based dye sensitizers using spin-component-scaled double-hybrid functionals: a TD-DFT benchmark study’ by Qabas Alkhatib *et al.*, *RSC Adv.*, 2022, **12**, 1704–1717, https://doi.org/10.1039/D1RA08795A.

In the original manuscript, the authors regret a misinterpretation in the results of the excitation energies calculated *via* the two double hybrid spin-component-scaled functionals DSD-BLYP and DSD-PBEP86 using ORCA version 4.2.0.

The authors originally stated that the excitation energies calculated *via* these two functionals are spin-component-scaled. The CIS(D) correction was computed for the DSD-BLYP and DSD-PBEP86 functionals for all dyes, resulting in excitation energies of double-hybrid quality. However, the spin-component scaling (SCS) and spin-opposite scaling (SOS) techniques were not applied to the CIS(D) correction.

The authors would like to indicate that the application of the SCS/SOS techniques to excited states has only been possible in ORCA version 5.0 and later, following the developments published by Casanova-Páez and Goerigk in July 2021.^1^ In ORCA 4.2 and earlier versions, spin-scaling in the DSD-BLYP and DSD-PBEP86 density functionals has been limited to ground-state calculations only.

The authors sincerely thank Lars Goerigk and his collaborators for clarifying this specific point, as the authors were previously unaware of this limitation.

An independent expert has considered and approved the corrected information.

The Royal Society of Chemistry apologises for any inconvenience to authors and readers.

## References

[cit1] Casanova-Páez M., Goerigk L. (2021). Time-dependent long-range-corrected double-hybrid density functionals with spin-component and spin-opposite scaling: a comprehensive analysis of singlet–singlet and singlet–triplet excitation energies. J. Chem. Theory Comput..

